# GAW20: methods and strategies for the new frontiers of epigenetics and pharmacogenomics

**DOI:** 10.1186/s12919-018-0113-1

**Published:** 2018-09-17

**Authors:** Nathan L. Tintle, David W. Fardo, Mariza de Andrade, Stella Aslibekyan, Julia N. Bailey, Justo Lorenzo Bermejo, Rita M. Cantor, Saurabh Ghosh, Philip Melton, Xuexia Wang, Jean W. MacCluer, Laura Almasy

**Affiliations:** 1 Department of Mathematics and Statistics, Dordt College, 498 4th Ave. NE, Sioux Center, IA 51250 USA; 2 Department of Biostatistics, University of Kentucky, 725 Rose St, Lexington, KY 40536 USA; 3 Division of Biomedical Statistics, Mayo Clinic, 200 First St. SW, Rochester, MN 55905 USA; 4 Department of Epidemiology, University of Alabama at Birmingham, 1655 University Blvd., Birmingham, AL 35205 USA; 5 Department of Epidemiology, Fielding School of Public Health, University of California, 650 Charles E. Young Dr. South, Los Angeles, CA 90095 USA; 6 Institute of Medical Biometry and Informatics, University of Heidelberg, Im Neuenheimer Feld 130.3, 69120 Heidelberg, Germany; 7 Department of Human Genetics, David Geffen School of Medicine at University of California, 650 Charles E Young Dr. South, Los Angeles, CA 90095 USA; 8 Indian Statistical Institute, 203 B T Rd., Kolkata, West Bengal 700108 India; 9 Curtin/UWA Centre for Genetic Origins of Health and Disease, School of Pharmacy and Biomedical Sciences, Curtin University and School of Biomedical Sciences, The University of Western Australia, 35 Stirling Hwy. (M409), Crawley, WA 6009 Australia; 10 Department of Mathematics, University of North Texas, 1155 Union Circle #311430, Denton, TX 76201 USA; 11 Department of Genetics, Texas Biomedical Research Institute, 8715 W. Military Dr., San Antonio, TX 78227 USA; 12 Department of Genetics, Perelman School of Medicine, University of Pennsylvania, 3400 Civic Center Blvd., Philadelphia, PA 19104 USA; 13 Department of Biomedical and Health Informatics, Children’s Hospital of Philadelphia, 3401 Civic Center Blvd., Philadelphia, PA 19104 USA

## Abstract

GAW20 provided a platform for developing and evaluating statistical methods to analyze human lipid-related phenotypes, DNA methylation, and single-nucleotide markers in a study involving a pharmaceutical intervention. In this article, we present an overview of the data sets and the contributions analyzing these data. The data, donated by the Genetics of Lipid Lowering Drugs and Diet Network (GOLDN) investigators, included data from 188 families (*N* = 1105) which included genome-wide DNA methylation data before and after a 3-week treatment with fenofibrate, single-nucleotide polymorphisms, metabolic syndrome components before and after treatment, and a variety of covariates. The contributions from individual research groups were extensively discussed prior, during, and after the Workshop in groups based on discussion themes, before being submitted for publication.

## Background

This supplement to *BMC Proceedings* contains the proceedings of the GAW20 that was held March 4–8, 2017, in San Diego, CA, USA. The GAWs were initiated in 1982 and have traditionally been held approximately biannually. They provide a discussion forum for developing and evaluating statistical methods aimed at deciphering the architecture of human complex diseases, mainly by identifying their genetic risk factors. Discussion and comparison of methods is facilitated by providing the same data sets to all researchers. These data sets are chosen by the GAW Advisory Committee, taking into consideration the suggestions and concerns of attendees. Discussion of future data sets begins the final day of the Workshop and remains open for at least a year. Data sets must be well characterized, address urgent needs for analysis tools in genetic epidemiology, and be available upon request prior to the Workshop. After the GAW organizers release the data sets, researchers analyze the data and prepare a manuscript to submit to the Workshop. All coauthors of submitted manuscripts are eligible to attend the Workshop. Active participation in group discussions is required, as well as attendance at overall presentation and discussion meetings. Individuals who provided data or participate in the Workshop organization may also attend. More information about GAW, including upcoming Workshops, may be found at http://www.gaworkshop.org.

## Genetic analysis workshop 20

GAW20 was the first GAW to explore the emerging field of epigenetic data, providing an opportunity to explore methodological questions of interest in epigenetics in the context of a family-based, longitudinal study that also included a pharmaceutical intervention. As with previous GAWs, analyses of these data by GAW20 participants largely focused on dealing with the high dimensionality of the single-nucleotide polymorphism marker data, accounting for the family structure and handling longitudinal data, with the new wrinkle of integrating DNA methylation data, all within the context of a clinical trial. These issues are natural considering the data set provided, which is described in detail in Aslibekyan et al. [1].

Although complete data set details are provided in Aslibekyan et al. [1], we provide a brief overview of the data set now as an introduction to this volume. Data from 188 families [*N* = 1105 individuals] participating in the Genetics of Lipid Lowering Drugs and Diet Network (GOLDN) study were the focus of analysis for GAW20. Data available on these 1105 individuals consisted of: (a) DNA methylation at 463,995 cytosine-phosphate-guanine (CpG) sites measured before and after a 3-week treatment with fenofibrate; (b) 906,000 single-nucleotide polymorphisms; (c) metabolic syndrome components ascertained before and after the drug intervention; and (d) relevant covariates. Methylation and genotype data were subject to a variety of standard filtering and quality control procedures. GAW20 participants had the option of focusing their methodological investigations on this “real” data or on an alternative version that was simulated (“simulated data”). Following a complex, but realistic, genetic model that hypothesized genetic modification of methylation on triglyceride levels at select loci, 200 replicates of simulated posttreatment methylation and triglyceride measurements were generated for each individual. Simulated data provided participants the opportunity to provide additional statistical validation and assessment of method performance.

The availability of the GAW20 data was announced by email in the fall of 2016 to roughly 3200 individuals on the GAW mailing list, resulting in 81 separate requests for data to participate in the Workshop. The number of GAW20 attendees in March 2017 was 80. Although individuals were allowed to present more analyses at the Workshop than had been described in their submitted papers, each group was still required to report the results of some analyses prior to the meeting to participate. Manuscripts were distributed among participants prior to the Workshop, and participants were assigned to discussion groups to facilitate discussion before and during the Workshop. Manuscripts from the other discussion groups were also available for download from the GAW20 online discussion forum or upon request prior to the Workshop. After the Workshop, 39 individual papers were accepted for publication and constitute this proceedings volume, with 12 papers accepted for publication in *BMC Genetics*.

Participants and contributions were from many countries, with the United States of America, Canada, and Germany providing the largest numbers of contributions. Additional contributing participants were from Australia, China, India, the United Kingdom, the Netherlands, Norway, Poland, Spain and Taiwan. The contributions were subdivided into 7 discussion groups by topic, with 1 group split into 3 subgroups to facilitate more detailed and focused discussions. The themes were Causal Modeling (Group 1), Data Mining and Machine Learning (Group 2), Epigenetics–Complex Models (Group 3a), Epigenetics–Gene Searching (Group 3b), Epigenetics–Longitudinal Analysis (Group 3c), GWAS (Genome-wide Association Studies) (Group 4), Genotype-by-Methylation (Group 5), Repeated Measures (Group 6), and Genetics of Treatment Response (Group 7). The papers in this proceedings volume are presented according to these groupings, with Groups 2 and 3a merged because of the overlapping goals of the papers in these groups. However, group assignment was often not easy and topics in groups may overlap. The contributed papers are preceded by the data description by Aslibekyan et al. [1] and a description of the model used to generate the simulated data by Kraja et al. [2]. Each group was led by a moderator with previous GAW experience. The moderator encouraged and organized the discussion and presentations prior to, during, and after the Workshop. Discussions largely started before the Workshop and continued at the Workshop within group meetings. Each discussion group, directed by the group leader, was also in charge of preparing a presentation of the issues discussed in the group and the conclusions drawn. These presentations were made to all GAW attendees in plenary sessions. There were also 2 poster sessions for presenting individual contributions. The Workshop closed with plenary sessions on lessons learned and planning for future GAWs. After the Workshop, the group leader was typically in charge of editing group manuscripts, as well as writing the summary paper for the group. To avoid possible conflicts of interest, articles to which the group editor contributed were reassigned to other groups for the editing process. Summary papers and individual papers deemed to be of highest impact are published in a supplement to *BMC Genetics*, and all other individual contributions are found in these proceedings.

Overall, GAW20 uncovered many new challenges and unsolved problems with epigenetic and pharmacogenomics data, although many of these challenges mirror those identified in the analysis of GWAS and whole-genome sequence data. The discussions highlighted the need for methodological development in almost all considered areas.
